# Cognitive sovereignty and decolonial public health: reclaiming epistemic authority in the global AI era

**DOI:** 10.3389/fpubh.2026.1785170

**Published:** 2026-03-26

**Authors:** Samuel Kakraba, Edmund Fosu Agyemang, Sudesh K. Srivastav

**Affiliations:** 1 Department of Biostatistics and Data Science, Celia Scott Weatherhead School of Public Health and Tropical Medicine at Tulane University, New Orleans, LA, United States; 2 Tulane Center for Aging, School of Medicine, Tulane University, New Orleans, LA, United States

**Keywords:** artificial intelligence ethics, cognitive sovereignty, decolonization, digital colonialism, global public health equity, Global South, health data governance, indigenous data sovereignty

## Abstract

As artificial intelligence (AI) becomes critical infrastructure for global health, it reproduces colonial patterns of extraction, mining data from the Global South to train models owned by the Global North. While international bodies like the WHO emphasize “ethical AI,” they often overlook the structural violence of this digital colonialism. This perspective argues that true health equity requires more than bias mitigation; it demands cognitive sovereignty: the right of communities to govern not just their data but also the epistemic logic, interpretive frameworks, and algorithmic reasoning of the systems that analyze it. Drawing from Indigenous data governance principles (OCAP/CARE) and concrete implementation cases from Kenya, Nigeria, Rwanda, and Latin America, we demonstrate how cognitive sovereignty extends beyond data sovereignty to encompass control over knowledge production itself. By anchoring this political vision in specific technical architectures, federated learning, and community-led surveillance, we can move from extractive “AI for good” to a decolonial future of autonomous health intelligence. Recent cases from Kenya's AI health deployments and pathogen genomics illustrate both the urgency and feasibility of this transformation.

## 1 Introduction

The narrative of AI in global health is seductive: predictive algorithms can track cholera in regions without laboratories, mobile apps can diagnose skin conditions where dermatologists are scarce, and large language models can assist clinicians in resource-limited settings. Indeed, artificial intelligence is revolutionizing many fields, including drug discovery through machine learning (ML)-enhanced quantitative structure-activity relationship (QSAR) modeling for identifying novel inhibitors of neurodegenerative disease targets and leukemia therapeutics (1), DNA polymerase inhibitor discovery for overcoming cisplatin resistance in cancer (2), AI-driven predictive diagnostics for Alzheimer's disease using handwriting analysis (3), automated AI pipelines for Parkinson's disease prediction via voice recordings (4), graph-theoretic modeling interfaced with unsupervised ML for analyzing sickle cell (5), cystic fibrosis (6) and SARS-CoV-2 spike protein mutations (7), ML-driven discovery of biologically significant associations among amino acids for protein science (8), aggregate interactome-based ML prediction of drug targets to limit protein aggregation in neurodegenerative diseases (9), among others. Recent evidence from Kenya, where an AI clinical copilot reduced diagnostic errors by 16% and treatment errors by 13% across nearly 40,000 patient encounters, appears to validate this promise (10, 11). Yet this optimism obscures a fundamental paradox. The very mechanisms that promise democratization, massive datasets, centralized cloud computing, and “universal” foundation models rely on a flow of raw material (data) from the South to the North that mirrors the extractive logic of 19th-century rubber, gold, and biological specimen trade (12).

Consider the Kenya AI Consult case more closely. While the trial demonstrated clinical efficacy, the underlying infrastructure reveals familiar colonial patterns: patient data from Nairobi clinics flowed to OpenAI's servers to train and refine models ultimately owned by a US corporation valued at over $150 billion. Local clinicians gained access to improved diagnostic support, but Kenya's health system gained no ownership of the model, no control over the algorithmic logic, and no share of the intellectual property or commercial value created from Kenyan patients' suffering. This is not a partnership; it is a high-tech iteration of the dependency structures that have characterized North-South relations for centuries (13, 14).

Recent high-level guidance, such as the WHO's January 2024 ethics frameworks for Large Multi-Modal Models, focuses heavily on “fairness,” “privacy,” and “bias mitigation” (15, 16). While necessary, these concepts are insufficient. They treat the symptoms of algorithmic harm (a biased output, a privacy breach) without challenging the structure of ownership (who owns the model, who profits from it, who controls its evolution). If a health system in Kenya relies on a diagnostic model hosted on servers in Seattle, trained on Kenyan data but patented by a US corporation, the system remains colonial regardless of how “unbiased” its predictions are. This is not hyperbole. Kenya's transformation into Africa's “Silicon Savannah” has been shadowed by troubling dependencies: 87% of smallholder farmers who once consulted community elders and indigenous weather signs now rely on algorithmic forecasts from platforms where the data they generate becomes proprietary to foreign entities (17). The farmers produce the intelligence; foreign corporations own insights.

## 2 From data sovereignty to cognitive sovereignty

We must escalate our demands from data sovereignty (control over inputs) to cognitive sovereignty (control over the reasoning process, interpretive frameworks, and knowledge production itself). Data sovereignty often effectively stops at the border: once data leaves a country to “help train” a global model, meaningful control is lost. Cognitive sovereignty, a concept gaining traction in Indigenous AI governance and emerging economy policy, asserts that a community must retain the ability to generate its own health insights using its own epistemology and interpretive logic (18, 19).

Cognitive sovereignty extends beyond the physical control of data (data sovereignty) to encompass three critical dimensions:

1. **Algorithmic autonomy:** the capacity to govern how health data is interpreted, what patterns are deemed significant, and which clinical logics are prioritized, not merely where data resides.
2. **Epistemic authority:** the right to determine which knowledge systems (Western biomedical taxonomies, indigenous syndromic surveillance, community-defined health signals) inform AI reasoning, rejecting the imposition of “universal” models that erase local clinical realities.
3. **Technological sovereignty:** the institutional capacity of Global South entities to develop, maintain, iterate, and innovate on AI infrastructure autonomously, rather than existing as perpetual recipients of Northern technology.

This framework builds on Indigenous data sovereignty principles, particularly the OCAP (Ownership, Control, Access, Possession) and CARE (Collective Benefit, Authority to Control, Responsibility, Ethics) principles developed by First Nations and the Global Indigenous Data Alliance (20–22). These frameworks operationalize sovereignty by distinguishing:

- **Ownership** (relationship to cultural knowledge and data) from **Possession** (physical stewardship);
- **Control** (authority over all aspects of data collection, use, and governance) from **Access** (ability to retrieve and use data);
- **Collective Benefit** (ensuring value flows back to communities) from mere consultation;
- **Authority** (binding decision-making power) from advisory participation.

Applied to health AI, cognitive sovereignty means communities must control not only where their data is stored but also the interpretive logic embedded in algorithms, the ownership structure of resulting models, and the governance mechanisms that determine how AI-derived insights are used, shared, and monetized (Table 1).

**Table 1:** Data sovereignty vs. cognitive sovereignty in health AI.

**Dimension**	**Data sovereignty**	**Cognitive sovereignty**
What it controls	Physical location and access to raw data.	Algorithmic reasoning, interpretive frameworks, model ownership, and knowledge production.
Governance scope	Data residency requirements, consent mechanisms, and cross-border flows.	AI design logic, training priorities, epistemic choices, IP ownership, benefit distribution.
Technical implementation	Data localization, encryption, and access controls.	Federated learning, community-governed model training, local compute infrastructure, and open weights.
Who decides	National data protection authorities, institutional ethics boards.	Communities, local health systems, regional AI governance collectives, and South-South networks.
Example violation	Kenyan health records transferred to US cloud without consent.	Diagnostic model trained on Kenyan data uses Western biomedical taxonomies exclusively, erasing local syndromic surveillance knowledge; the model is patented by a US corporation; Kenya has no IP rights or governance role.
Redress mechanism	Data repatriation, fines for breaches.	Model co-ownership, benefit-sharing agreements, community veto power over deployment, algorithmic reparations.

### 2.1 Epistemic violence through technological universalism

Technological universalism, the assumption that a model trained on Western electronic health records works as a “global” baseline, is a form of epistemic violence (23, 24). It erases local clinical realities and devalues indigenous knowledge systems. Diagnostic algorithms often prioritize Western biomedical taxonomies over local syndromic surveillance methods that have protected communities for generations. For instance, algorithms designed for pneumonia detection in high-income settings fail to account for malaria-pneumonia co-presentation patterns prevalent in sub-Saharan Africa, leading to missed diagnoses and inappropriate treatment (25). Facial recognition systems used in dermatology AI demonstrate lower accuracy for darker skin tones because training datasets are skewed toward lighter-skinned populations (26). These are not technical glitches; they are manifestations of whose knowledge counts and whose bodies matter in the design of supposedly “universal” systems.

Cognitive sovereignty rejects this erasure, demanding that AI systems function as “students” of local knowledge rather than “teachers” of foreign norms. It insists that communities retain authority to define health signals, validate clinical patterns, and determine which algorithmic outputs align with lived epidemiological experience. When communities in Uganda use citizen report cards to track health service quality, resulting in higher child immunization rates and decreased mortality, they exercise cognitive sovereignty: defining their own metrics, interpreting their own data, and demanding accountability on their own terms (27, 28).

### 2.2 Protecting against reverse surveillance

This shift from data to cognitive sovereignty also protects against reverse surveillance: the use of health data and AI-derived insights as tools for geopolitical monitoring and control of vulnerable populations (29). When AI systems are developed and owned externally, health data becomes intelligence that can be weaponized by state or corporate actors against the communities that generated it (30). Consider Nairobi's AI-powered surveillance system, processing footage from approximately 1,800 cameras: despite significant public investment, the algorithm sourcing, accuracy benchmarks, and data retention policies remain opaque, implemented through government-to-government agreements with minimal public consultation. Digital lending platforms in Kenya determine creditworthiness for millions using proprietary algorithms with no meaningful explanation for loan rejections and little recourse for contesting decisions. These systems extract knowledge from populations, then use that knowledge to classify, predict, and control them, a contemporary iteration of colonial surveillance infrastructures (31).

Cognitive sovereignty ensures that surveillance serves local needs first and remains accountable to the people being monitored. It means communities retain veto power: if a proposed AI deployment cannot demonstrate how it strengthens local autonomy or if it risks reverse surveillance, communities have binding authority to block it.

### 2.3 Architecture as politics: a decolonial blueprint

A decolonial stance is empty without technical implementation. Two specific architectures offer pathways to operationalize cognitive sovereignty, transforming it from aspiration to infrastructure.

#### 2.3.1 Federated learning as sovereign infrastructure

Federated Learning (FL) is typically marketed as a privacy-preserving feature, but its political potential is far greater. In FL architecture, the model travels to the data, learns from it locally, and returns only aggregated mathematical insights, not individual patient records. This reverses the colonial flow. Instead of raw data moving North, the algorithm moves South, submits to local training, and leaves the raw wealth (data) in its place of origin (32, 33).

To facilitate understanding across disciplines, a brief overview of how Federated Learning works and its main types is provided here. The FL process follows an iterative cycle of five core steps: (1) a coordinating server initializes a global model and distributes it to participating institutions (e.g., hospitals or health agencies); (2) each institution trains the model locally on its own data without sharing raw patient records; (3) only the learned model updates—such as mathematical parameters or gradients—are sent back to the coordinating server; (4) the server aggregates these updates (commonly using an algorithm called Federated Averaging) to produce an improved global model; and (5) the updated model is redistributed to participants, and this cycle repeats until the model achieves satisfactory performance (32, 33). Crucially, at no point does sensitive health data leave the institution where it originates.

There are three principal types of Federated Learning, each suited to different data-sharing scenarios. Horizontal Federated Learning (HFL) applies when multiple institutions hold datasets with the same types of information (features) but on different individuals. For example, several hospitals may each record age, blood pressure, diagnosis, and lab results for their own patients. HFL allows these hospitals to collaboratively train a shared model without pooling their patient records. Vertical Federated Learning (VFL) applies when different organizations hold different types of information about the same individuals. For instance, a hospital may hold clinical records while an insurance provider holds claims data for the same patients; VFL enables a joint model that leverages both data sources without either party revealing its raw data. Federated Transfer Learning (FTL) addresses situations where participating institutions differ in both their patient populations and the types of data they collect. FTL leverages knowledge from a well-resourced institution to improve model performance at institutions with limited data, enabling cross-domain collaboration (34). In public health applications, HFL is the most commonly used architecture—employed, for example, in multi-hospital disease surveillance and epidemic modeling—while VFL is increasingly used for cross-sector analyses that integrate clinical, socioeconomic, and behavioral data sources.

More critically, FL enables technological sovereignty: the capacity of Global South institutions to host compute infrastructure, govern training processes, and participate as equal partners rather than data donors. Recent systematic reviews of federated learning in public health document its technical feasibility for disease surveillance, outbreak prediction, and clinical decision support across resource-limited settings. However, implementation challenges remain significant: high computational costs for local nodes, governance complexities in multi-stakeholder networks, and regulatory harmonization across jurisdictions (34).

These challenges are not insurmountable; they are political. If Global South data improves a Northern model's performance by 20%, should that translate to 20% computational access, 20% of revenue, or 20% ownership of intellectual property? Cognitive sovereignty demands we ask and answer this question structurally, not charitably.

Global health funders must fundamentally redirect investment. Instead of building massive central data lakes that consolidate power in Northern institutions, funding should prioritize:

- **Local compute capacity:** GPU clusters, edge servers, and cloud infrastructure hosted in low-resource settings, enabling them to operate FL nodes as equal partners with governance authority, not merely as data sources.
- **South-South FL networks:** regional federated architectures connecting African CDC member states, Latin American indigenous health initiatives, and Asian LMIC health systems, bypassing Northern intermediaries entirely (35, 36). The African Union's AI strategy and ASEAN's digital health frameworks already prioritize regional sovereignty (37); FL provides technical infrastructure to realize these visions.
- **Open-weight models and governance protocols:** ensuring that models trained on federated data remain accessible to originating communities, with transparent governance over model updates, deployment decisions, and intellectual property (38).

Precedents exist. The H3Africa consortium established regional biorepositories in West, East, and South Africa, creating distributed infrastructure for genomic research with data governance committees led by African scientists (39, 40). The same model applies to AI: federated nodes governed locally, coordinated regionally, interfacing globally on equitable terms.

#### 2.3.2 Community-led surveillance (CLS)

Traditional surveillance is extractive and top-down: it harvests information from communities to report upward to national or global bodies, often with minimal feedback or local benefit. Community-Led Surveillance (CLS), empowered by simple digital tools, inverts this logic: intelligence stays local first.

When communities define their own “signals”: water quality changes, unusual fever patterns, environmental threats, they own the early warning system. AI should amplify communities' capacity to detect and respond to health threats before external aid arrives, rather than extracting signals for global dashboards that communities never see (41, 42). This shifts AI's role from “monitoring the poor” to “equipping the frontline”.

CLS is not merely an ethical preference; it is epidemiologically superior. Extensive evidence demonstrates that community-led approaches detect outbreaks earlier, improve response times, and strengthen local accountability compared to top-down surveillance systems:

- **Uganda:** citizen report cards tracking health service quality contributed to higher child immunization rates, decreased under-5 mortality, reduced health worker absenteeism, and shorter clinic wait times (27, 28, 43).
- **Tanzania:** the AfyaData smartphone app enabled One Health disease surveillance by community health workers, detecting zoonotic disease risks and environmental health threats that formal systems missed (44).
- **Lesotho:** participatory surveillance via weekly phone calls during COVID-19 provided real-time epidemiological intelligence from communities, informing rapid local responses (45).
- **Nigeria:** the Community Health Tracking Initiative in Lagos implemented community-defined health priorities rather than imposing vertical disease tracking from national capitals. Community-oriented health efforts increasingly emphasize locally defined priorities, with health workers, community leaders, and residents collaborating to identify indicators such as malnutrition, flood-related illnesses, and psychosocial distress so that monitoring reflects the lived health needs of communities rather than only external donor agendas (46). Data remains community-controlled; national health ministries access insights through transparent data-sharing agreements with community vetoes.
- **India:** in India, participatory epidemiology and community-based surveillance initiatives have trained local community members to collect and interpret health data, using culturally meaningful syndromic descriptions that link symptoms to local seasons and contexts, rather than relying solely on biomedical categories developed in Euro-American settings (47).
- **Africa-wide:** community-led monitoring (CLM) coalitions for HIV, TB, and malaria documented human rights violations, improved service availability and quality, and created enabling policy environments through inclusive stakeholder engagement (48).

#### 2.3.3 Mechanisms of digital colonialism: why ethical AI frameworks fail

The gap between ethical AI principles and decolonial practice reflects a deeper structural problem. Major AI frameworks focus on reducing harm within existing power structures rather than redistributing power itself (49). They ask, “How can we make this model less biased?” rather than “Who owns this model, and who benefits from it?”.

##### 2.3.3.1 The apartheid of health data ownership

This is evident in how data flows across borders. Health systems in the Global South actively consent to share data with Northern corporations and research consortia, often under pressure from funders or partnership terms, without understanding the long-term intellectual property implications or benefit-sharing mechanisms that will never materialize (50, 51). The apartheid in global health AI is not hidden; it is embedded in standard practice. Models trained in suffering and biological samples of Global South populations are owned, patented, and commercialized by Northern entities, while communities receive neither attribution nor compensation nor meaningful access to the resulting technologies.

Consider pathogen genomics: during the Ebola outbreak, viral samples from West African patients were rapidly sequenced and shared with international databases, enabling scientific breakthroughs and vaccine development (52). Yet African institutions had minimal ownership of resulting intellectual property, limited access to sequencing infrastructure, and little voice in governance of the genomic data they provided. This pattern repeated during COVID-19, despite decades of advocacy for equitable benefit-sharing (53). As one African genomics leader noted, “The trend that relegates researchers from developing countries to the role of sample collectors instead of equal partners and custodians of the samples and data must stop.” (39).

Similarly, Kenya's agricultural AI platforms harvest data from hundreds of thousands of smallholder farmers, creating valuable datasets for insurers, lenders, and agritech companies, yet farmers neither own the insights nor share in the profits. In healthcare, AI diagnostic systems developed using Pfizer's ATLAS database (containing over 850,000 antimicrobial resistance samples from 83 countries, including Kenya) are used by Kenyan doctors, yet the underlying database and intellectual property remain proprietary to Pfizer (17).

##### 2.3.3.2 Colonial sanitary conferences redux

This pattern echoes the colonial sanitary conferences of the 19th century, when health became a vector for geopolitical control and resource extraction. Today's language of “closing the digital divide,” “leaving no country behind in the fourth industrial revolution,” and “frictionless data transfer” represents contemporary iterations of developmentalism, the same rhetorical framework used to justify colonialism itself (54, 55). The digital colonialism of today operates through consent and partnership language, making it harder to recognize and resist.

WHO's 2024 guidance on LMMs, while comprehensive in technical recommendations, dedicates minimal attention to ownership structures, benefit-sharing mechanisms, or power redistribution. It urges governments to “invest in public infrastructure” and developers to “engage diverse stakeholders,” but stops short of demanding that Global South institutions co-own models trained on their data or that communities retain veto authority over deployments. This is reform, not transformation. It seeks to make extraction ethical, rather than to end extraction.

## 3 Discussion

### 3.1 Toward a decolonial framework for public health AI

Implementing decolonial principles in global health AI requires structural changes across multiple domains, moving beyond advisory recommendations to binding governance mechanisms.

#### 3.1.1 Algorithmic reparations and binding benefit-sharing

Communities must own, control, and benefit from the health data they generate, with enforceable mechanisms and non-voluntary guidelines. The concept of algorithmic reparations builds on precedents from genomics benefit-sharing and historical redress cases:

**The HeLa precedent:** in 2023, descendants of Henrietta Lacks reached a historic settlement with Thermo Fisher Scientific, securing compensation (terms confidential) for the company's decades-long commercialization of HeLa cells taken without Lacks' consent in 1951. While the settlement came 70 years later, it establishes a critical principle: retroactive justice for data extraction is possible. Entities that profit from biological or digital material obtained without consent or equitable terms can be held accountable (56, 57).

**Genomics benefit-sharing frameworks:** the H3Africa consortium developed comprehensive benefit-sharing frameworks for pathogen genomics research, identifying benefits across micro (individual), meso (institutional), and macro (societal) levels, including capacity building, co-authorship, infrastructure development, and health system improvements. Key principles include fairness, reciprocity, transparency, proportionality, and community authority to define what constitutes meaningful benefit (39, 40).

Operationalizing algorithmic reparations:

- **Performance-linked benefit sharing:** if Global South data improves a commercial model's accuracy by X%, that percentage of resulting revenue, computational access, or equity ownership, it must automatically flow back to the originating health system or community. This should be contractually binding and auditable, not voluntary corporate social responsibility.
- **Intellectual property co-ownership:** models trained substantively on Global South data must list originating institutions as co-inventors on patents, with proportional rights to licensing revenue and decision-making authority over model use.
- **Open-weight requirements:** publicly funded models, or those relying on Global South data, must release model weights under open licenses that guarantee free access for originating communities, even if commercial use requires licensing fees.
- **Retroactive audits:** AI companies currently profiting from Global South health data should face mandatory third-party audits to quantify contributions from LMIC datasets, with reparations mechanisms (revenue sharing, infrastructure investment, capacity building) proportional to documented extraction.

#### 3.1.2 Federated and decentralized architecture as default

Investment must shift fundamentally toward local AI training, local control, and data infrastructure that keeps sensitive health information sovereign. This requires building technological capacity in Global South institutions, not as recipients of Northern charity, but as autonomous developers and innovators.

**Funding conditionality:** major funders, including the Gates Foundation, Wellcome Trust, NIH, and USAID, should reject grant proposals that centralize Global South data in Northern repositories. Preferential funding with higher award amounts and expedited review must go to projects using decentralized, federated, or edge-computing architectures that guarantee data residency and local governance authority.

**Infrastructure investment:** funding should prioritize:

- Building and maintaining local compute clusters (GPUs, high-performance servers) in LMIC institutions.
- Training data scientists and AI engineers from Global South countries, with retention incentives to prevent brain drain.
- Establishing regional AI governance bodies (e.g., African CDC AI coordination, Latin American indigenous AI networks) with binding authority over regional federated systems.
- Open-source AI tooling adapted for low-bandwidth, intermittent connectivity environments.

Kenya's fiber optic infrastructure model offers a template: 65% industry investment, 15% multinational participation, and 20% government stake, ensuring local oversight while leveraging external capital (17). This model can scale data center and AI compute infrastructure and maintain sovereignty while accessing necessary resources.

#### 3.1.3 Co-design with community veto authority

AI systems affecting health must be shaped by the communities they serve, not solely by technologists and data scientists. This means recognizing community members as experts in their own health contexts and embedding their knowledge in system design from inception.

Communities must move from “consultation” (advisory input with no binding power) to “veto authority” (binding decision-making power):

- If a proposed AI deployment cannot demonstrate how it strengthens local cognitive sovereignty, communities must have enforceable power to block it.
- If algorithmic outputs conflict with community knowledge or lived epidemiological experience, communities can demand model retraining or reject deployment;
- If an AI system risks reverse surveillance or extractive data use, communities have legal standing to halt it.

**Precedents exist:** the Black Health Equity Working Group developed the EGAP (Equitable Genomics Access Protocol) framework, establishing community governance tables where Black communities exercise decision-making authority and not just consultation over genomic data use. ^112^ Indigenous data governance frameworks (OCAP/CARE) operationalize community veto through data access committees and culturally appropriate consent protocols.

#### 3.1.4 Decolonial health diplomacy and south-south solidarity

Power in global health AI governance must shift to Global South leadership. This requires rejecting extractive “partnership” models and fostering genuine South-South collaboration (58).

**Examples:**

- **African CDC federated AI capacity:** building continent-wide federated learning infrastructure for disease surveillance, governed by African institutions, interfacing with global networks as equal partners rather than data sources.
- **Latin American indigenous AI initiatives:** UNESCO's work with indigenous communities in Latin America to develop culturally grounded AI systems that center indigenous languages, worldviews, and knowledge systems (59).
- **South-South technology transfer:** Brazil-Nigeria partnerships in space technology and agriculture; SADC-ALBA health collaboration; BRICS AI working groups prioritizing local ownership and technology sovereignty (35, 36, 60).

These initiatives demonstrate that the Global South (representing 85% of humanity and 120+ countries) possesses collective negotiating power to demand structural change. The challenge is coordination, not capacity.

### 3.2 Implications and call to action

Global health institutions face a defining choice. Will AI become a tool for continued extraction and control, or can it be redirected toward liberation and equity? This is not merely a technical question; it is fundamentally political and decolonial.

The burden of change cannot rest solely on communities or Global South actors. Journals, funders, and health institutions in the Global North must actively platform decolonial voices, amplify community-led research and surveillance, and critique the digital colonialism embedded in mainstream AI-for-health discourse.

#### 3.2.1 Journals: establishing accountability standards

Journals must establish editorial standards requiring that health AI studies disclose:

- Data governance structures (who owns data, who controls access, and what consent was obtained).
- Model ownership (who holds intellectual property rights, what licensing terms apply);
- Benefit-sharing agreements (what material benefits, if any, flow back to data-originating communities or institutions);
- Authorship equity (Are Global South researchers listed as co-authors or relegated to acknowledgments despite providing data?) (61).

Journals should actively solicit and prioritize research by Global South authors on locally led AI development and reject papers that treat Global South populations as data sources without co-authorship or governance roles. This is not “lowering standards”; it is correcting structural bias that privileges Northern institutions with more resources to analyze data extracted from the South.

#### 3.2.2 Funders: conditioning grants on sovereignty protections

Funders must condition funding on demonstrated data sovereignty protections and community governance mechanisms:

- Grant proposals centralizing Global South data in Northern repositories should face automatic rejection or require extraordinary justification.
- Preferential funding (higher amounts, faster review) should reward federated architecture, local computer investment, and binding benefit-sharing.
- Post-award audits should verify that promised sovereignty protections are implemented, with funding clawback for non-compliance.

Funders must also invest substantially in building compute capacity and AI talent in Global South institutions, treating this not as “capacity building” (a term implying deficiency) but as investment in innovation hubs that will advance global health in ways Northern institutions cannot.

#### 3.2.3 Health institutions and consortia: rewriting partnership agreements

WHO, Gates-funded initiatives, academic medical centers, and global health consortia must rewrite partnership agreements to include:

- Mandatory benefit-sharing (revenue sharing, co-ownership, free computational access) proportional to data contributions.
- Data residency requirements ensuring sensitive health data remain within originating countries unless federated architectures with local governance are used.
- Community veto power allowing local health systems or communities to halt AI deployments that violate sovereignty principles or create unacceptable risks.

Institutions must also systematically audit existing AI deployments for signs of digital colonialism (centralized Northern ownership, extractive data flows, lack of benefit-sharing) and commit to transitions toward federated or community-led alternatives within defined timeframes (e.g., 3–5 years).

## 4 Geographic scope and emerging models: beyond Kenya

While Kenya's AI clinical copilot is instructive, cognitive sovereignty must be demonstrated across diverse contexts.

### 4.1 Africa's health data governance infrastructure

**Rwanda:** Rwanda's National Health Intelligence Center, launched in April 2025, aggregates real-time health data from facilities and community health workers nationwide to enable AI-driven analytics, disease surveillance, and policy decisions while supporting data sovereignty in regional contexts (62). This positions Rwanda as a model for cognitive sovereignty without isolation.

**Burkina Faso:** Burkina Faso's National Community Health Strategy embeds digitization at its core, with community health workers (ASBCs) co-creating roadmaps for digital tools that integrate local data collection into national systems while ensuring community-responsive surveillance reflects villager-defined priorities (63).

**South Africa:** South Africa's Wits University Reproductive Health and HIV Institute leads regional research on federated learning for tuberculosis and HIV data analysis, enabling collaborative AI model training across Southern African institutions without centralizing patient data and exemplifying South-South health data partnerships (64).

### 4.2 Asia-pacific and Latin American models

**India:** the All India Institute of Medical Sciences' participatory epidemiology program scales community-led surveillance across rural India while preserving local disease classification systems. India's recent National Health Stack prioritizes data residency within India while exploring federated learning for multistate disease surveillance (65).

**Thailand:** the Thailand Digital Health Ecosystem explicitly incorporates local knowledge systems such as Thai traditional medicine and community-based health worker expertise into AI model design through federated learning architectures. This prevents the epistemic dominance of Western biomedical categories (66).

**Brazil:** Brazil's Unified Health System (SUS), one of Latin America's most comprehensive public health infrastructures, supports community-participatory health surveillance in urban vulnerable areas like favelas, where residents help prioritize monitoring for local issues such as dengue outbreaks, violence-related injuries, and mental health challenges (67).

These examples demonstrate cognitive sovereignty is not an abstract ideal but an emerging organizational model, actively implemented across the Global South.

## 5 The stakes for global health

By taking these actions, institutions can catalyze a paradigm shift to one where AI becomes a tool for health sovereignty, not domination. The HeLa settlement demonstrates that retroactive justice, even decades after extraction, is achievable. Community-led surveillance in Uganda proves that local epistemic authority produces superior epidemiological outcomes. H3Africa shows that equitable genomic research infrastructure can be built and governed by African institutions. Federated learning provides technical architecture to operationalize data sovereignty at scale.

The obstacles to cognitive sovereignty are not technically political. What is required is the collective will to redistribute power, to center Global South leadership, and to recognize that genuine health equity in the AI era demands nothing less than epistemic liberation. The alternative is a future where health intelligence remains a 21st-century commodity extracted from the Global South to enrich the Global North, perpetuating colonial hierarchies under the banner of “innovation.” The future of equitable global health depends on which path we choose.

## Author reflexivity statement

As researchers based at a US institution, SK, EA, and SS recognize the inherent tensions of writing about decolonial health AI from within Northern academia. This perspective is grounded in sustained engagement with scholarship by Global South researchers, Indigenous data sovereignty frameworks, and consultations with colleagues in Kenya, Nigeria, Ghana, India, Rwanda, Thailand, Brazil, Burkina Faso, South Africa, and across Latin America.

The authors affirm that cognitive sovereignty must ultimately be defined and led by Global South communities themselves; this piece is intended to amplify those voices and propose technical pathways for implementation, not to speak on behalf of communities.

## Data Availability

The original contributions presented in the study are included in the article/supplementary material, further inquiries can be directed to the corresponding author.
